# Use of bioengineered human acellular vessels to treat traumatic injuries in the Ukraine–Russia conflict

**DOI:** 10.1016/j.lanepe.2023.100650

**Published:** 2023-05-06

**Authors:** Oleksandr Sokolov, Vasyl Shaprynskyi, Oleh Skupyy, Oleksandr Stanko, Serhii Yurets, Yuliya Yurkova, Laura E. Niklason

**Affiliations:** a Dnipro State Medical University, Dnipro, Ukraine; b State Institution of Science Research and Practical Center of Preventive and Clinical Medicine, Kyiv, Ukraine; c Vinnytsya Regional Clinical Hospital, Vinnytsya, Ukraine; d Odessa Regional Clinical Hospital, Odessa, Ukraine; e Humacyte Global, Inc., NC, USA

On February 24th 2022, the Russian Federation invaded Ukraine. Consistent with other modern conflicts, blasts and shrapnel penetration have caused a large fraction of the traumatic injuries, creating contaminated wounds that often compromise the vascular supply to limbs and organs. Although autogenous veins are the preferred option for repair, their limited availability leads to the use of synthetic grafts. However, usage of synthetic vascular grafts carries a high risk of infection, meaning that there is a lack of suitable conduit in some war-injured patients.

Human Acellular Vessels (HAVs) are bioengineered vascular conduits that are cultured from human donors’ smooth muscle cells and subsequently decellularized.^1^ The HAV is an investigational biological product in late-stage clinical development, with over 1,000 patient-years of exposure. After clinical implantation, the HAV repopulates with cells, producing a living vascular tissue that may be highly resistant to infection.^2^^,^^3^ In March 2022, surgeons in Ukraine requested the HAV for vascular repair under Humanitarian conditions. In response, the manufacturer of the HAV (Humacyte Global Inc.) worked with the International Office of the US FDA and the Ukrainian Ministry of Health to provide HAVs to five hospitals in Ukraine. Surgeons in Ukraine were trained remotely, by video conferencing, on the procedures applicable to the HAV.

Since June 2022, 13 patients who lacked autologous vein for repair have been treated with the HAV to repair a range of arteries including superficial femoral, common femoral, popliteal, and brachial arteries. Of these, 11 sustained limb vascular injuries in the ongoing conflict, mostly comprising blast and shrapnel wounds. One such patient is shown in Fig. 1, who underwent repair of the common femoral artery on day 1, had patency of the HAV confirmed at day 52, and who began ambulating on day 119.

**Fig. 1:**
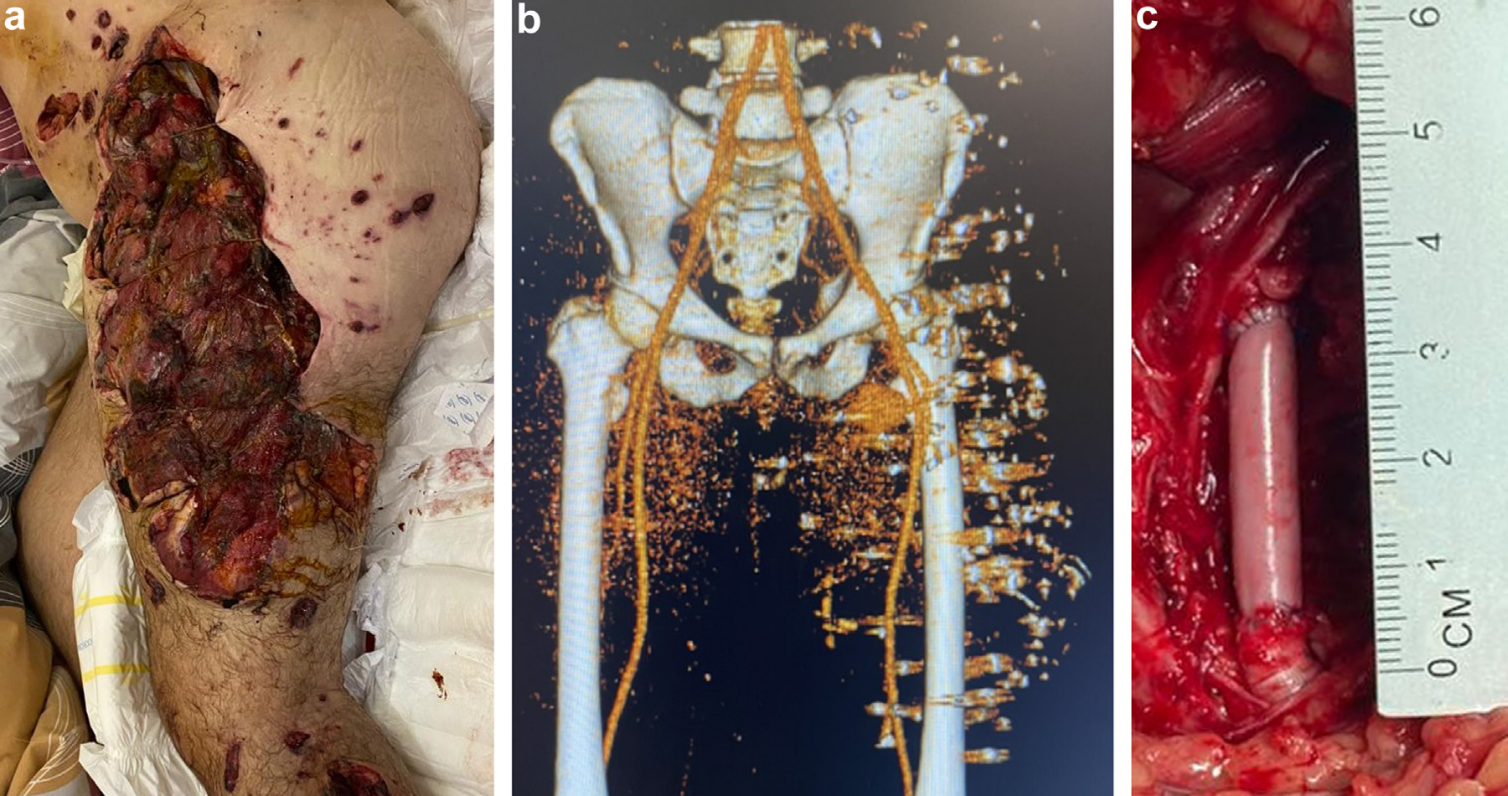
**War-wounded patient in Ukraine.** a. 29-year-old male suffered blast injury to the left leg; b. Pre-operative CT scan shows shrapnel fragments, including in vascular bundle near inguinal ligament; c. Repair of left common femoral artery with HAV, which has resisted infection during follow-up.

Three patients received HAVs after failure of either saphenous vein (n = 2) or synthetic graft (n = 1) to repair an arterial injury. As of January 29th 2023, all HAVs retained primary patency, and no infections nor amputations of the affected limbs were reported. After follow-up times ranging from 1 to 7 months, there have been no reports of HAV conduit infection or mechanical failure. The Ukraine real-world trauma experience demonstrates the potential for regenerative medicine technologies to improve patient outcomes in resource-limited environments.

## Contributors

LEN: Conceptualization, data curation, formal analysis, funding, resources, supervision, writing – original draft, and writing – review & editing.

OSo: Participated in data collection, data analysis, data interpretation, methodology, project administration, supervision, and writing – review & editing.

OSk: Participated in data collection, data analysis, data interpretation, methodology, project administration, supervision, and writing – review & editing.

OSt: Participated in data analysis, data interpretation, methodology, project administration, supervision, and writing – review & editing.

VS: Participated in data collection, data analysis, data interpretation, methodology, project administration, supervision, and writing – review & editing.

SY: Participated in data collection, data analysis, data interpretation, methodology, project administration, supervision, and writing – review & editing.

YY: Participated in resources, data collection, data analysis, data interpretation, methodology, project administration, supervision, and writing – review & editing.

## Data sharing statement

Since this is a Humanitarian aid program, data will only be made available for research purposes under certain conditions, and upon specific written request and justification to the Corresponding Authors.

## Role of funding source

The biologics (HAV Human Acellular Vessel) were provided to the Ukrainian surgeons in the context of Humanitarian Aid.

## Ethics committee approval

The Humacyte aid to Ukraine surgeons and patients was approved under a Humanitarian Aid Program procedure by the Ukraine ministry of health and the relevant local institutions.

## Declaration of interests

LEN: LEN is the founder and CEO of Humacyte, has stock, is a Board member, and receives salary from Humacyte. LEN performed most of the writing of the first draft and the subsequent drafts of this Correspondence. LEN is the inventor on multiple patents that are either owned by Humacyte, or licensed by Humacyte, has an Officer and fiduciary role at Humacyte in her capacity as CEO, and has stock and options in Humacyte.

YY: YY is an employee of Humacyte and has stock.

VS: VS has no conflict of interest to disclose.

OSk: OS has received honoraria from Bayer and Servier in the past 36 months; no conflict of interest for this manuscript.

SY: SY has received honoraria from Bayer and Servier in the past 36 months; no conflict of interest for this manuscript.

OSo: OSo has no conflict of interest to disclose.

OSt: OSt has no conflict of interest to disclose.

## References

[1] Niklason L.E., Lawson J.H. (2020). Bioengineered human blood vessels. Science.

[2] Lawson J.H., Glickman M.H., Ilzecki M. (2016). Bioengineered human acellular vessels for dialysis access in patients with end-stage renal disease: two phase 2 single-arm trials. Lancet.

[3] Kirkton R.D., Santiago-Maysonet M., Lawson J.H. (2019). Bioengineered human acellular vessels recellularize and evolve into living blood vessels after human implantation. Sci Transl Med.

